# Supplementary material for: Iterative improvement in the automatic modular design of robot swarms
Source: PeerJ Comput Sci. 2020 Dec 7;6:e322. doi: 10.7717/peerj-cs.322 (PMC7924708; doi:10.7717/peerj-cs.322)
Supplement: Supplemental Information 3 [file peerj-cs-06-322-s003.zip › argos3/doc/api/standalone/pages.html]

ARGoS: Page Index


- Main Page
- Related Pages
- Namespaces
- Classes
- Files

# Related Pages

Here is a list of all related documentation pages:

- Todo List

---

Generated on 10 Jul 2018 for ARGoS by 
 1.6.1
